# Uncovering global metabolic response to cordycepin production in *Cordyceps militaris* through transcriptome and genome-scale network-driven analysis

**DOI:** 10.1038/s41598-018-27534-7

**Published:** 2018-06-18

**Authors:** Nachon Raethong, Kobkul Laoteng, Wanwipa Vongsangnak

**Affiliations:** 1 Interdisciplinary Graduate Program in Bioscience, Faculty of Science, Kasetsart University, Bangkok, 10900 Thailand; 2 Bioprocess Technology Laboratory, Food Biotechnology Research Unit, National Center for Genetic Engineering and Biotechnology (BIOTEC), National Science and Technology Development Agency (NSTDA), Pathum Thani, 12120 Thailand; 3 Department of Zoology, Faculty of Science, Kasetsart University, Bangkok, 10900 Thailand; 4 Computational Biomodelling Laboratory for Agricultural Science and Technology (CBLAST), Faculty of Science, Kasetsart University, Bangkok, 10900 Thailand

## Abstract

The cellular metabolic adaptations of *Cordyceps militaris* have been progressively studied. In particular, the cordycepin pathway is of interest in medicinal applications. Even though the metabolic pathways for cordycepin production are known to be related to different carbon sources, the regulatory mechanisms at a systems level are poorly characterized. To explore the regulatory mechanisms, this study therefore aimed to investigate the global metabolic response to cordycepin production in *C. militaris* through transcriptome analysis and genome-scale network-driven analysis. Here, transcriptome analysis of 16,805 expressed genes in *C. militaris* strain TBRC6039 grown on different carbon sources was performed. Of these genes, 2,883 were significantly differentially expressed genes, uncovering sucrose- and glucose-mediated changes in the transcriptional regulation of central carbon metabolism in *C. militaris*, which was shown using the CmSNF1 mechanism as an example. After applying genome-scale metabolic network-driven analysis, reporter metabolites and key metabolic subnetworks involving adenosine, cordycepin and methionine were proposed through the up-regulation of cordycepin biosynthetic genes. Our findings suggest that the transcriptional regulation of these pathways is a ubiquitous feature in response to specific culture conditions during cordycepin overproduction.

## Introduction

*Cordyceps militaris*, belonging to a group of entomopathogenic fungi in the phylum Ascomycota, has long been used in folk medicine and therapy in Asia. The great potential for medicinal usage of this fungus is generally attributed to its bioactive metabolites, e.g., cordycepin, ergosterol, mannitol and exopolysaccharide^1^. Cordycepin or 3′-deoxyadenosine, which is derived from adenosine by the absence of oxygen at the 3′ position of its ribose backbone, is a unique compound found in *C. militaris*^2^. It displays potent cytotoxicity against several types of cancer cells. As such, it has been used as a chemical marker for quality control in this fungal species^3^. In addition to the important role of cordycepin in drug development for cancer treatment, several bioactive compounds produced by *C. militaris* also exhibit beneficial properties, including anti-oxidant, anti-microbial, anti-inflammatory, and anti-metastatic effects and immune enhancement^1^. This variety of biological functions has attracted much attention in pharmaceutical applications and thus contributes to the commercial value of fungal products^4^. Because of its slow growth in nature, there is awareness of the over-exploitation of wild *C. militaris* in terms of species extinction at the population level and even extinction of the whole species in the ecosystem^1^. As such, several efforts have been made to investigate efficient strains, cost-effective culture media and cultivation conditions to increase the biomass and productivity of specialty metabolites of *C. militaris*^5–7^. Nevertheless, the biotechnological production of whole-cell mass and targeted compounds, particularly cordycepin, remains challenging in the commercial sector.

Generally, carbon sources are directly linked to the biosynthesis of several metabolites in fungal cells. The growth characteristics of *C. militaris* on various carbon sources have been studied^5^. It has been documented that hexose, a six-carbon (C6) sugar, such as glucose, was the favorable carbon source for cordycepin production due to the relation of high sugar uptake and cordycepin accumulation^5^. The disaccharide sugar, particularly sucrose, was also a preferred carbon source for practical large-scale production of cordycepin^7^. Additionally, a comparative study of two entomopathogenic fungi, *C. militaris* and *Cordyceps sinensis*, in submerged cultivations demonstrated that sucrose was the best carbon source for mycelial biomass and exopolysaccharide productions in both species^8^. Very recently, an intensive study on the cultivation of *C. militaris* strain TBRC6039 demonstrated that although xylose was less favorable than glucose or sucrose in terms of biomass productivity, this particular strain could efficiently metabolize xylose based on the observation that the five-carbon (C5) sugar was exhausted during cultivation^9^. Further analyses of key metabolites also showed that a high production yield of cordycepin in *C. militaris* strain TBRC6039 was obtained when it was grown in xylose rather than in favorable carbon sources (i.e., glucose and sucrose)^9^. These results suggested that *C. militaris* strain TBRC6039 might contain alternative gene(s)/pathway(s) for metabolizing xylose, even though it lacks the bacterial-like xylulose-5-phosphate phosphoketolase gene, *xpkA*^10^. Noticeably, xylose and phosphoribosyl pyrophosphate (PRPP), which are important precursors for *de novo* purine biosynthesis, share the same five-carbon ring in their molecular structures. Therefore, most of the assimilated xylose in this strain might be channeled towards the biosynthetic pathway of cordycepin rather than towards central carbon metabolism^11^. Possibly, the alteration of a sole carbon source might lead to metabolic adaptations in central carbon metabolism for cell growth and other relevant metabolic processes.

Emerging omics tools and analyses have been exploited to explore the cell behavior of *C. militaris*. Complete sequencing of the *C. militaris* genome has provided a large number of open reading frames; however, some open reading frames have unknown functions^12^. In addition, several genome-scale data sets from previous studies are publicly available, but only a few insightful studies on transcriptional regulation systems in *C. militaris* exist^13,14^. Current transcriptome analysis, in conjunction with advanced RNA sequencing (RNA-Seq) technology^15^ and bioinformatics infrastructure, has been one of the most promising approaches for the identification of responsive genes, their regulatory modes and relevant transcription factors in acclimatization to specific biotic and abiotic factors during a metabolic shift. This information has been enriched through not only RNA-Seq technology, but also systems biology tools, such as genome-scale metabolic networks, which can be used as a scaffold for integrated analysis with multi-level omics data, permitting the systematic identification of reporter metabolites that represent hot spots in terms of metabolic regulation^16^. This approach would underpin the core biological processes governing carbon utilization in *C. militaris*. So far, even though the metabolic pathways involved in cordycepin production are known to be tied to different carbon sources, the regulation of cellular mechanisms at the systems level is poorly characterized.

This study therefore aimed to investigate the global metabolic response of *C. militaris* strain TBRC6039 in cordycepin production on different carbon sources, i.e., sucrose, glucose and xylose, through transcriptome analysis and genome-scale network-driven analysis. RNA-Seq data for *C. militaris* strain TBRC6039 was obtained through Illumina sequencing technology and further processed through transcriptome assembly, annotation and assessment. By integrating transcriptome analysis and genome-scale network-driven analysis, the global metabolic response to cordycepin production in *C. militaris* in specific culture conditions was uncovered. This study provides useful information on the systems-wide overproduction of cordycepin in *C. militaris*, which is valuable knowledge in industrial biotechnology.

## Results and Discussion

### Growth characteristics and cordycepin production of *C. militaris* on different carbon sources

The growth characteristics of *C. militaris* on sucrose, glucose and xylose are presented in Table 1. These results show that sucrose was a preferred carbon source for cell growth as indicated by the maximum specific growth rate (μ_max_) and high biomass productivity. A reduction in growth rate on glucose was observed relative to growth on sucrose (Table 1). Possibly, individual molecules of glucose and fructose can enter central carbon metabolism for biomass formation, leading to enhanced biomass production in sucrose culture. In contrast, xylose was an unfavorable carbon source for *C. militaris* growth because its maximum specific growth rate was the lowest (0.10 ± 0.04 day^−1^) relative to the other carbon sources tested. The poor growth rate found in xylose culture agreed with the earlier substrate utilization profiles of *C. militaris* and other insect pathogenic fungi, e.g., *Beauveria bassiana* and *Nomuraea rileyi*^10^. Mycelial growth was faster in sucrose culture than in other carbon sources, and 2.5-fold increase was obtained in sucrose culture compared to the xylose culture; however, once the cordycepin content was considered, the different sources of carbon offered remarkably different productivity of extracellular cordycepin. This result is possibly because some carbon sources preferably served as precursors for a wide range of primary metabolites and secondary metabolites that are important for growth and metabolite production, respectively^17^. The highest titer of cordycepin was found in the medium broth with xylose at 149.5 ± 15.71 mg/L, whereas titers of 119.32 ± 14.06 mg/L and 109.14 ± 11.54 mg/L were obtained in sucrose and glucose cultures, respectively. Notably, the enhanced extracellular cordycepin titer in xylose culture was obtained by prolonged cultivation (Table 1), which is in agreement with a previous report^18^. However, this phenomenon was not found in glucose and sucrose cultures. Nevertheless, the extracellular cordycepin productivity of the xylose culture was lower than that of the sucrose and glucose cultures (Table 1). These results are consistent with those of other reports on various *C. militaris* strains^7,19^. *In vitro C. militaris* cultivation in distinct sole carbon sources could be used to accelerate biological studies in addition to *in vivo* biological processes^10^. Using the optimal condition for *in vitro* culture, the cultivation time can be shortened to obtain maximal biomass and cordycepin production and enable the recapitulation of developmental stages at transcriptional and translational levels^14^.

**Table 1:** Growth characteristics and cordycepin production of *C. militaris* using different carbon sources.

Features	Sucrose	Glucose	Xylose
Maximum specific growth rate, μ_max_ (day^−1^)	0.25 ± 0.05^a^	0.19 ± 0.02^a^	0.10 ± 0.04^b^
Biomass productivity^c^ (g/L/day)	0.21 ± 0.01^a^	0.23 ± 0.03^a^	0.06 ± 0.01^b^
Extracellular cordycepin titer (mg/L)	119.32 ± 14.06^a,b^	109.14 ± 11.54^a^	149.50 ± 15.71^b^
Extracellular cordycepin productivity^d^ (mg/L/day)	5.97 ± 0.70^a^	5.46 ± 0.41^a^	2.49 ± 0.21^b^

^a,b^Different superscript letters in rows indicate statistically significant differences (p-value ≤ 0.05, Tukey’s test). All presented data of the growth characteristics were the highest values of individual cultures. Value is mean ± SD (n = 3).

^c^The highest biomass productivities of sucrose, glucose and xylose cultures were obtained from the cultivations at 16, 14 and 60 day, respectively.

^d^The highest extracellular of cordycepin productivities of sucrose, glucose and xylose cultures were obtained from the cultivations at 20, 20 and 60 day, respectively.

### Transcriptome data assembly, annotation and assessment for *C. militaris*

**Figure 1:**
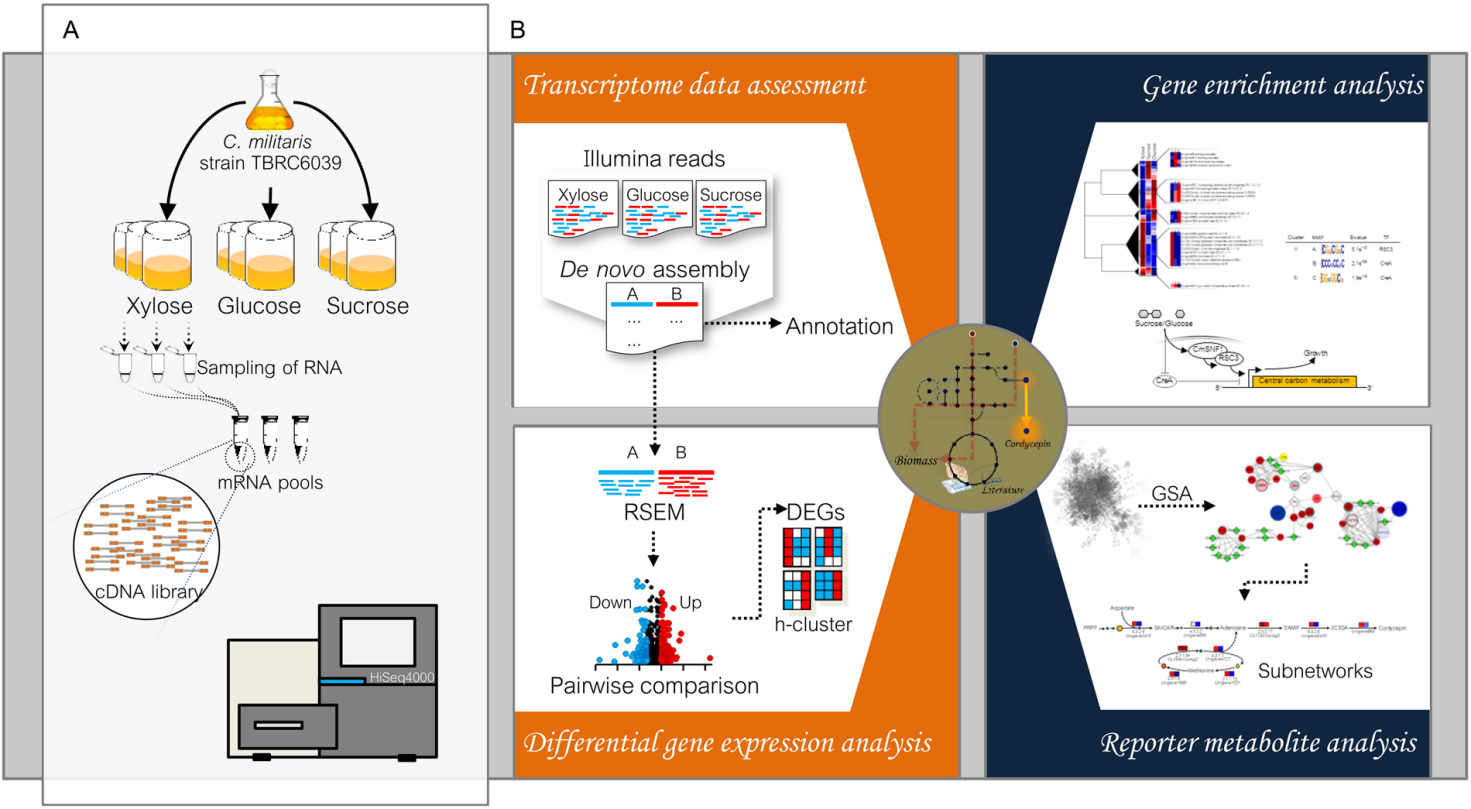
Systematic workflow of transcriptome analysis and genome-scale network-driven analysis. (**A**) Cultivation process *via* transcriptome data generation using an Illumina HiSeq. 4000 sequencer. (**B**) Transcriptome data assessment through differential gene expression analysis across different carbon sources and subsequent gene clustering and reporter metabolite analysis.

As different growth conditions induced by various carbon sources result in altered phenotypes, it was of interest to investigate transcriptional changes in relation to *C. militaris* metabolic behavior. A schematic overview of the experimental setup from the cultivation process towards transcriptome assessment and genome-scale network-driven analysis is illustrated in Fig. 1. *C. militaris* mRNA pools isolated from sucrose, glucose and xylose cultures were sequenced using an Illumina HiSeq. 4000 sequencer. As a result, raw reads at an average sequencing depth of 47.51 million paired-end reads were gained. After removing adaptor and low-quality sequences and read pollution, clean reads were finally retrieved with an average sequencing depth of 44.97 million paired-end reads and a sequencing quality of 98.69%, 98.84% and 98.77% for sucrose, glucose and xylose cultures, respectively (Table 2). Once all clean reads obtained from each carbon source were combined, a large fraction of 13.49 Giga base pairs (Gbps) was consequently obtained and processed through a Trinity-assembling pipeline^15^. This step resulted in 16,805 expressed genes, which comprises 10,838 unigenes (i.e., singleton genes) and 5,967 CL-genes (i.e., genes with different isoforms).

**Table 2:** Assembled *de novo* transcriptome statistics.

Features	Sucrose	Glucose	Xylose
Sequencing depth (million paired-end reads)	44.87	45.15	44.88
Sequencing quality (%)	98.69	98.84	98.77
Number of expressed genes	16,640	16,396	16,601
Total number of expressed genes (Total number of protein sequences)	16,805 (13,851)		

**Figure 2:**
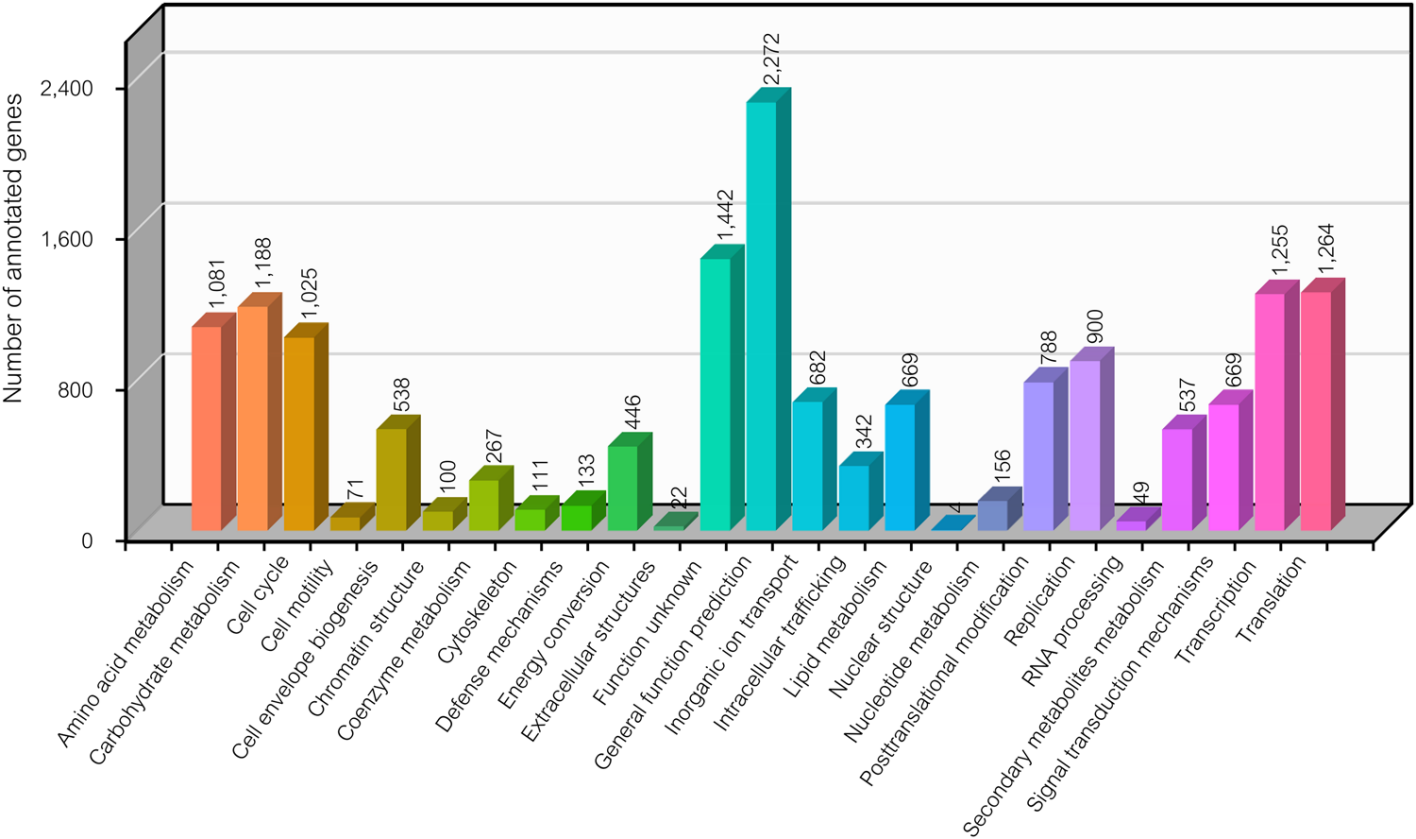
Distribution of different functional categories in *C. militaris* transcriptome using the COGs database.

Regarding the annotation of all 16,805 expressed genes, 13,851 genes were identified as protein-encoding genes, which were composed of 13,771 genes according to NR, SwissProt, GO, COGs, InterPro and KEGG databases^20–25^ and 80 genes according to ESTScan detection^26^. For functional assignment of the 13,851 protein-encoding genes, functions could be predicted for more than 98% of the genes based on different protein databases, i.e., COGs (7,720 genes), KEGG Orthology (KO) (10,267 genes), GO (9,464 genes) and NR (13,209 genes). Supplementary File 1 lists annotated genes and putative functions according to the different protein databases used in this study. In Fig. 2, an example of various functional categories of genes based on the COGs database is illustrated. Among the 25 functional categories across 7,720 genes, the major categories were associated with metabolic functions (3,578 genes), which involves the metabolism of carbohydrates (1,188 genes), amino acids (1,081 genes), inorganic ion transport (682 genes), lipids (669 genes), secondary metabolites (537 genes), energy conversion (446 genes), coenzymes (267 genes) and nucleotides (156 genes) (see Fig. 2 and Supplementary File 1). In agreement with the COGs analysis, metabolism represented the majority of gene functions, accounting for 4,395 out of 10,267 genes and 5,763 out of 9,464 genes based on KEGG identifiers and GO terms, respectively (see Supplementary File 1).

**Figure 3:**
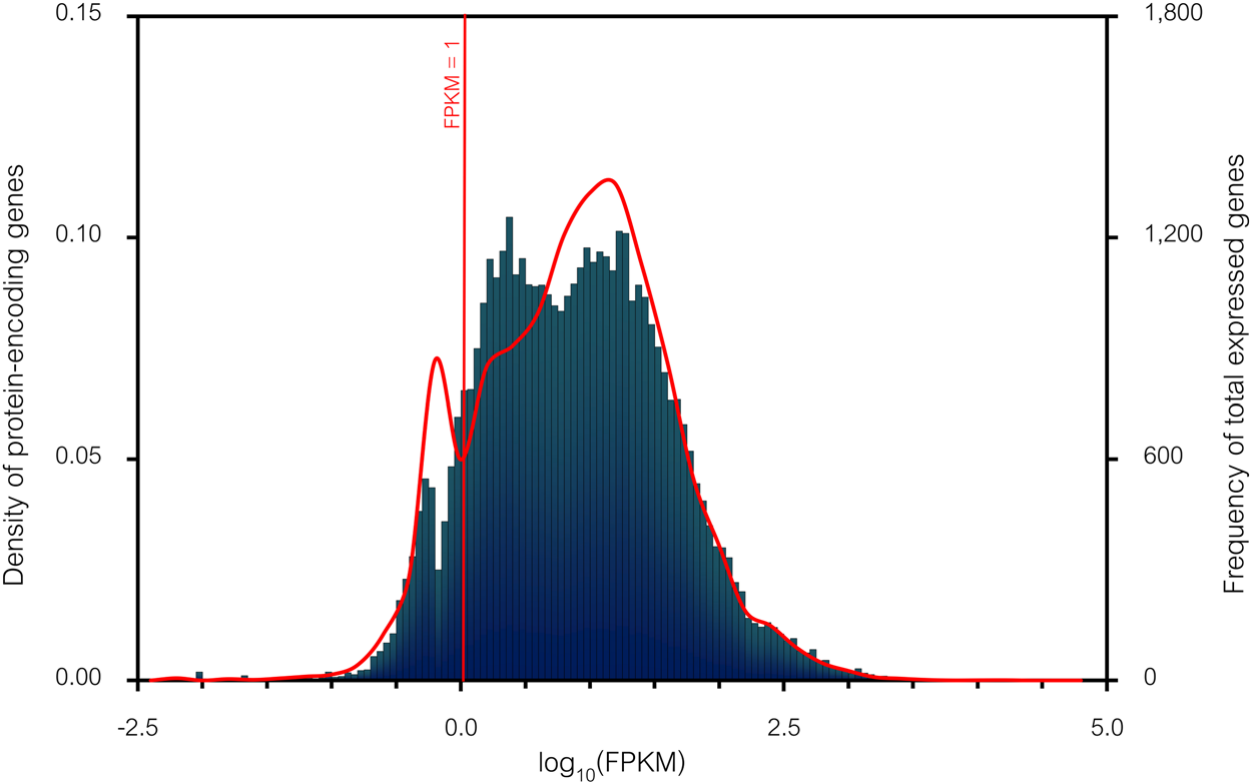
The FPKM distribution against the density of protein-encoding genes and frequency of total expressed genes. Red line graph shows the FPKM distribution against the density of protein-encoding genes. Bar chart shows the FPKM distribution against the frequency of total expressed genes. Dashed line represents the FPKM under a cut-off of 1.0 (log_10_ (FPKM) = 0.0).

**Figure 4:**
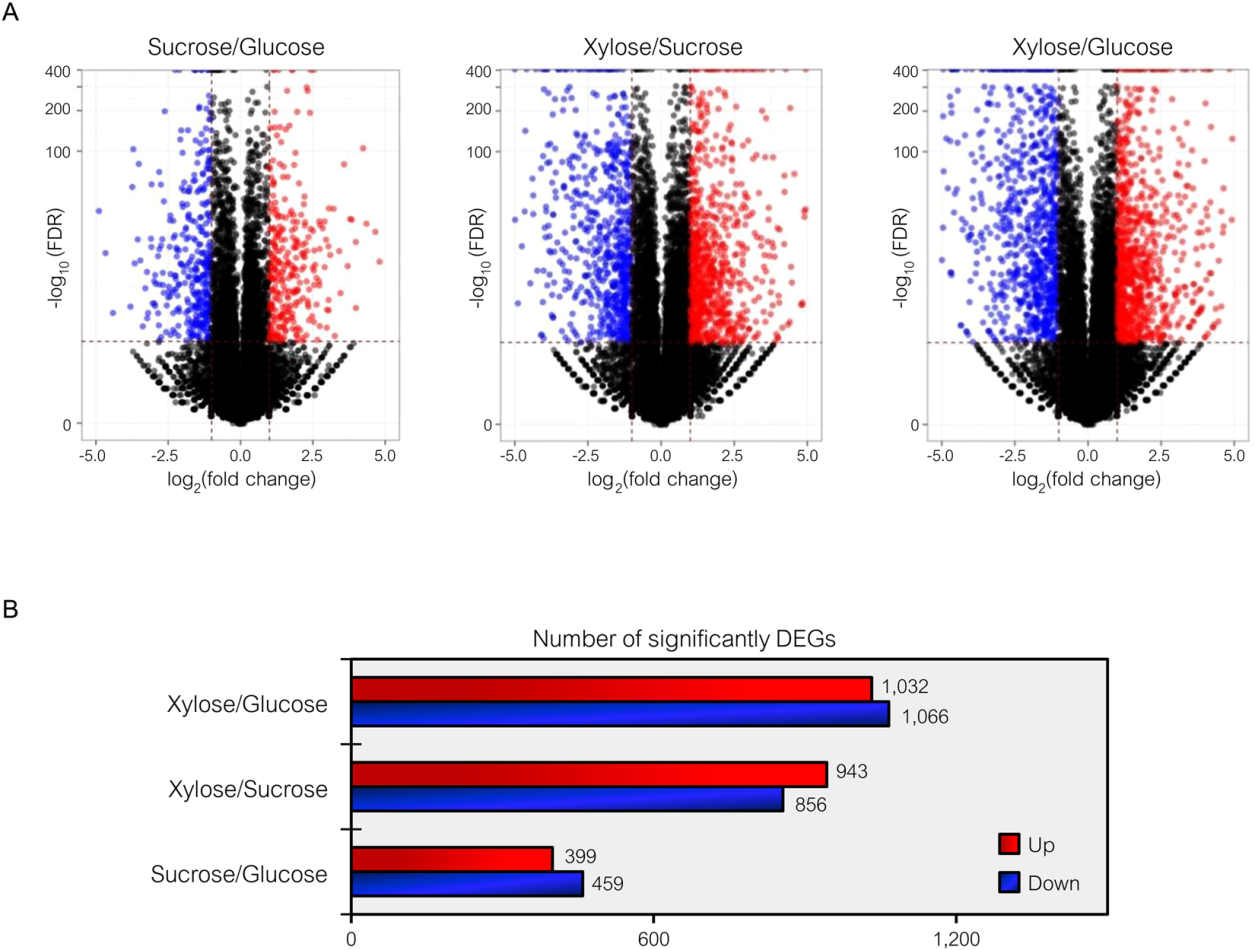
Differentially expressed gene analysis across pairwise carbon source comparisons. (**A**) Volcano plots show the DEGs under −log_10_ (FDR) against the log_2_ (fold change) between pairwise carbon source comparisons. The red and blue dots represent significantly up- and down-regulated expressed genes (FDR ≤ 0.001), respectively. Black dots are not significant DEGs. (**B**) Horizontal bar chart shows the number of significant DEGs in each pairwise comparison set.

Apart from the previously described annotations of all expressed genes, the relative expression abundances in the form of fragments per kilobase of transcript per million mapped reads (FPKM) distribution (see Methods) were also calculated for each gene. Figure 3 shows the correlation patterns between the FPKM distribution (log_10_ (FPKM)) against the frequency of total expressed genes (16,805 genes, bar chart) and the density of protein-encoding genes (13,851 genes, red line graph). Under a threshold of FPKM ≥ 1.0, most of the expressed genes were subjected to further analysis of differentially expressed genes (DEGs) between possible pairwise carbon source comparisons as shown in Fig. 4A.

### Differentially expressed gene analysis across pairwise carbon source comparisons

Transcriptome data were organized into three pairwise comparisons: sucrose versus glucose, xylose versus sucrose, or xylose versus glucose. Under the thresholds of |log_2_ (fold change)| ≥ 1 with a false discovery rate (FDR) ≤ 0.001 between possible pairwise carbon source comparisons, the number of significantly DEGs was identified as illustrated in Fig. 4B, and these genes were distributed into sucrose versus glucose condition (858 genes), xylose versus sucrose condition (1,799 genes) and xylose versus glucose condition (2,098 genes). Considering the removal of redundant genes identified from any pairwise carbon source comparisons, 2,883 significant DEGs were identified (Supplementary File 2). It is clear that the number of significant DEGs between the sucrose and glucose comparison was much less than the number of significant DEGs in other pairwise carbon source comparisons (Fig. 4B). This finding might be related to the fact that sucrose is a dimer of glucose and fructose.

To further understand the transcriptional regulation of central carbon metabolism in growth and biomass production in *C. militaris*, a number of significant DEGs (2,883 genes) related to GO terms were used to search the consensus gene set enrichment analysis results and identify any specific biological processes related to carbon source alterations. When sucrose and glucose cultures were compared, it was clearly seen that the up-regulated genes in sucrose metabolism were enriched in the sucrose condition, such as CL1855.Contig2, which encodes invertase (beta-fructofuranosidase, EC: 3.2.1.26). These results have been reported in a similar fashion when sucrose was utilized in yeast^27,28^. Importantly, beta-fructofuranosidase is a key carbohydrate-active enzyme used for the hydrolysis of sucrose into its derivatives, such as fructo-oligosaccharide, which is commonly used as a prebiotic in the pharmaceutical and functional food industries^29^. Considering the other pairwise carbon source comparisons, i.e., xylose versus glucose and xylose versus sucrose, it is obvious that the down-regulated genes in malate metabolic processes, chitin catabolism, carbohydrate and amino acid metabolism and transport were enriched in xylose culture (see Supplementary File 3). In contrast, most of the up-regulated genes on xylose were associated with fatty acid oxidation, phospholipid metabolism, carbohydrate phosphorylation, inositol biosynthetic process and galactose metabolism.

### Uncovering major changes in the transcriptional regulation of central carbon metabolism mediated by carbon source signaling

**Figure 5:**
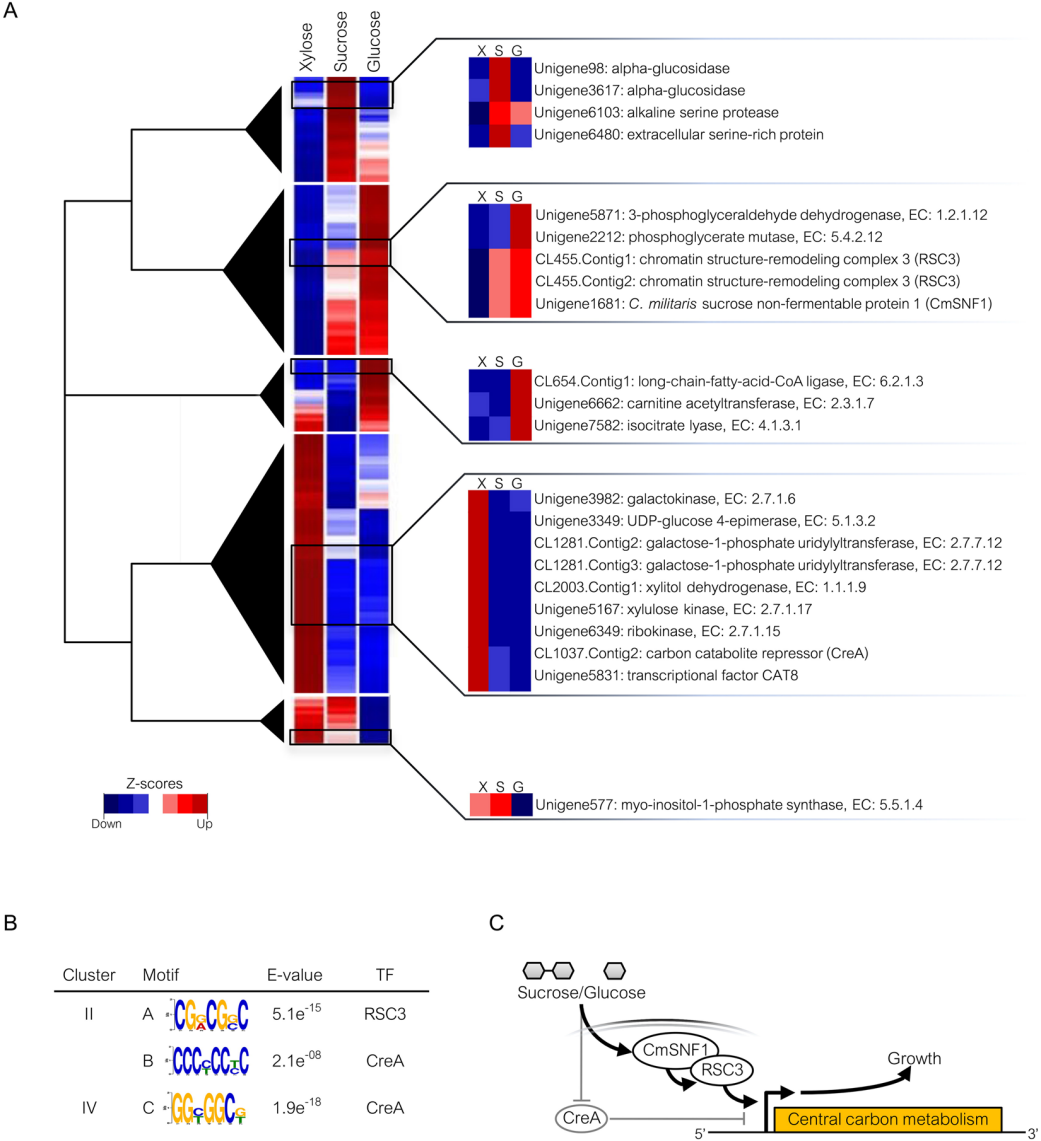
A proposed regulatory model of carbon sources signaling mediated transcriptional responses in central carbon metabolism of *C. militaris* revealed by clustering analysis and transcription factor identification. (**A**) Heat map diagram shows different gene clusters across three carbon sources comparison. The representative genes and their functions are listed on the right of heat map. Each gene is colored by the normalized expression values (Z-scores) across different carbon sources. (**B**) Putative DNA motifs and transcription factors (TF) are involved in transcriptional regulation of central carbon metabolism. (**C**) A proposed regulatory model of sucrose- and glucose-mediated major changes in transcriptional regulation of central carbon metabolism in *C. militaris* as an example shown in regulation of CmSNF1 mechanism.

To uncover how carbon source signaling mediated major changes in the transcriptional regulation of central carbon metabolism, hierarchical clustering analysis was implemented to determine the relative expression patterns among three carbon sources, i.e., sucrose, glucose and xylose. The correlation of significant DEGs revealed five clusters based on their expression patterns (Fig. 5A and Supplementary File 4). It is remarkable that the transcriptional profiles of genes encoding alpha-glucosidase (Unigene98 and Unigene3617), alkaline serine protease (Unigene6103) and extracellular serine-rich protein (Unigene6480) were up-regulated and denoted in Cluster I when sucrose was utilized as a sole carbon source. This finding might suggest a signature for sucrose utilization by *C. militaris*. Alkaline serine protease belongs to a family of subtilisin-like enzymes involved in entomopathogenic infection and the transition between saprophytic and parasitic stages. There was evidence of increased expression of several extracellular proteolytic enzymes, particularly serine protease, when the fungus was in close contact with an insect host. For instance, up-regulation of the protein degrading enzymes of the insect pathogenic fungus *Metarhizium anisopliae* was observed in an artificial medium supplemented with insect cuticle and hemolymph^30^ as complex carbon sources. Additionally, it appeared that a non-reducing disaccharide of glucose, i.e., trehalose, was the major source of carbon and energy in the circulating hemolymph of most insects^31^. Relating trehalose together with the other carbon sources, e.g., sucrose and raffinose, these additional sources served as not only substrates for insect growth, but also an autophagy inducer for macromolecules by degrading enzyme production in keratinocytes^32,33^. In contrast, the expression of specific genes (e.g., alkaline serine protease in Cluster I) significantly decreased in the presence of simple monosaccharide, such as glucose or xylose.

The occurrence of up-regulated expression patterns of genes encoding common enzymes that catalyze the steps of glycolysis, such as 3-phosphoglyceraldehyde dehydrogenase, EC: 1.2.1.12 (Unigene5871) and phosphoglycerate mutase, EC: 5.4.2.12 (Unigene2212), was identified in Cluster II when glucose was utilized as a sole carbon source. This finding indicates that glucose was primarily mediated as part of the transcriptional change in the central carbon metabolism of *C. militaris*. Similarly, the ubiquitous expression of *C. militaris* sucrose non-fermentable protein 1 (CmSNF1) encoded by Unigene1681 was apparently activated when glucose or sucrose was used as a sole carbon source (Fig. 5A). It has been reported that mutation of a CmSNF1 ortholog resulted in impaired conidial formulation in *B. bassiana*^34^ and failure to undergo pseudohyphal differentiation in response to nutrient limitation in yeast^35^. Moreover, specific genes, e.g., CL455.Contig1 and CL455.Contig2 (Cluster II), that encode chromatin structure-remodeling complex 3 (RSC3) were up-regulated during the activation of CmSNF1. This protein has been reported to play an important role in cell wall integrity and is critical for cell expansion during growth and morphogenesis in the fungus^36^.

Regarding Cluster III, the up-regulation of genes encoding enzymes involved in fatty acid metabolism, such as long-chain-fatty-acid-CoA ligase, EC: 6.2.1.3 (CL654.Contig1) and carnitine acetyltransferase, EC: 2.3.1.7 (Unigene6662), was identified in glucose culture. This up-regulation might mediate the toxicity of excess glyoxylate arising from the metabolism of fatty acids^37^. It is thus possible that an increased expression level of isocitrate lyase, EC: 4.1.3.1 (Unigene7582) might trigger the catalysis of the first reaction in the glyoxylate shunt to detoxify glyoxylate during glucose utilization.

Surprisingly, many genes were enriched in Cluster IV, encoding for the enzymes involved in galactose metabolism, including UDP-glucose 4-epimerase, EC: 5.1.3.2 (Unigene3349); galactokinase, EC: 2.7.1.6 (Unigene3982); and galactose-1-phosphate uridylyltransferase, EC: 2.7.7.12 (CL1281.Contig2 and CL1281.Contig3). The genes were up-regulated when xylose was utilized as a sole carbon source. This result suggests galactose metabolism as a possible metabolic target of glucose repression. Moreover, up-regulation of xylitol dehydrogenase, EC: 1.1.1.9 (CL2003.Contig1); xylulose kinase, EC: 2.7.1.17 (Unigene5167); and ribokinase, EC: 2.7.1.15 (Unigene6349) was observed (Cluster IV). These findings demonstrate that *C. militaris* could metabolize xylose as an alternative carbon source through the xylose metabolic pathway. The transcription factors in Cluster IV, such as regulatory protein CAT8 (Unigene5831) and carbon catabolite repressor CreA (CL1037.Contig2), were also up-regulated in xylose culture. For the other gene cluster (Cluster V), interestingly, Unigene577 encoding myo-inositol-1-phosphate synthase (EC: 5.5.1.4) was found. This enzyme catalyzes the rate-limiting step in inositol biosynthesis and was clearly up-regulated to drive cell growth under glucose limitation conditions^38^.

As previously mentioned, the differential gene expression patterns achieved with carbon source alteration (Fig. 5A) raised an interesting biological question as to how transcription factors are involved in the regulation of global metabolic responses. To explore this question, the promoter sequence of each gene in an individual cluster was examined for DNA motif identification, and the results are shown in Fig. 5B. Expectedly, the RSC3-like motif (motif A, CGRCGSC) was mostly identified in the promoter sequences of genes in only Cluster II, whereas CreA-like motifs, i.e., motif B (CCCYCCYC) and motif C (GGYGGCK), were found in the promoter sequences of genes in Cluster II and IV, respectively. It is noted that although the CmSNF1-like motif was not found in the promoter sequences of genes, the CmSNF1 gene, which was up-regulated in Cluster II, might trigger differential gene expression levels. This change might activate genes involved in glucose and sucrose metabolism that govern cell growth. In addition, CmSNF1 might recruit transcriptional activator RSC3 to access its motif site, thereby activating transcriptional changes, which might occur as reported for the crosslinking mechanism of SNF and RSC complex in yeasts^39^. Noticeably, in the CreA-like motif, this finding might suggest that the up-regulation of CreA on xylose as a sole carbon source could lead to the down-regulation of genes involved in other carbon utilization conditions, e.g., glucose, as shown in Cluster IV. Based on these overall observations, a regulatory model of carbon source signaling-mediated transcriptional responses in *C. militaris* central carbon metabolism is proposed (Fig. 5C).

### Improved annotation through genome-scale network reconstruction for integrated transcriptome analysis of *C. militaris*

Comparative gene analysis between *C. militaris* strain TBRC6039 (13,851 genes) and strain Cm01 (9,651 genes)^12^ was performed for annotation improvement. Pairwise gene sequence comparisons showed that many homologous genes were identified, accounting for 85.6% of genes (11,862 genes). After performing a taxonomic mapping of *C. militaris* strain TBRC6039 using KEGG, the results clearly showed that the majority of sequences matched existing genes in other Ascomycetes, particularly in entomopathogenic fungi belonging to the Cordycipitaceae, Ophiocordycipitaceae and Clavicipitaceae families. Interestingly, the matched genes and enzyme functions were obviously involved in common pathways found in entomopathogenic fungi, including carbohydrate metabolism, nucleotide metabolism, nutrient uptake and secondary metabolite biosynthesis, e.g., glycosidases, 5′-nucleotidase (EC: 3.1.3.5), adenylosuccinate synthetase (EC: 6.3.4.4), oligopeptide transporter, methyltransferases and polyketide synthases. Notably, *C. militaris* strain TBRC6039 had a high copy number of genes encoding chitinase (EC: 3.2.1.14) and nitrogen assimilation transcription factor NirA, which are remarkable proteins in the metabolism of insect pathogens^10^. Moreover, it had a high copy number of transport genes involved in nutrient and sugar uptake, such as gamma-aminobutyric acid (GABA) permease and monosaccharide transporters. These findings may relate to the ability of *C. militaris* strain TBRC6039 to assimilate nutrients from surrounding environments, such as the larvae body and culture medium.

Improved annotation data were incorporated into the metabolic reactions in the latest version of the genome-scale network of *C. militaris* (*i*WV1170)^11^ to enhance the network reconstruction. During the reconstruction process, 147 additional metabolic reactions were selected based on the expression of the included enzyme-encoding genes. For instance, two reactions involved in arginine and proline metabolism were added by L-ornithine N^5^-monooxygenase (EC: 1.14.13.196), and another four reactions involved in glyoxylate and dicarboxylate metabolism were added by hydroxypyruvate reductase, EC: 1.1.1.81 (2 reactions); glyoxylate reductase, EC: 1.1.1.79 (1 reaction); and methylglyoxal reductase, EC: 1.1.1.283 (1 reaction). Even though adenosine is a well-known direct precursor of cordycepin, the existing pathway for cordycepin biosynthesis in *i*WV1170 was limited because it was missing the intermediate reactions that bridge the gap between adenosine and cordycepin.

Beyond improving the genome annotation through this study, a novel gene cluster containing five genes, encoding oxidoreductase domain-containing protein (Unigene960), phosphoribosylamino-imidazole-succinocarboxamide (SAICAR) synthase (Unigene2410), ATP phosphoribosyltransferase (CL1730.Contig1 and CL1730.Contig2), and putative transporter (Unigene610), were identified as involved in the biosynthesis of cordycepin in *C. militaris* strain TBRC6039. This finding agrees with four physically linked genes (Cns1, Cns2, Cns3 and Cns4) that were recently determined to be a single gene cluster responsible for the biosynthesis of cordycepin in *C. militaris*^40^. This novel gene cluster and the nine corresponding reactions were introduced into the network to enhance the biosynthetic pathway of cordycepin.

In terms of connecting reactions, transport reactions, which have functional roles in transporting substances, e.g., sugars, amino acids, organic acids and ions, between cellular compartments (i.e., extracellular space, plasma membrane, cytosol, mitochondria, and peroxisome), were considered in the enhanced metabolic network reconstruction. According to the expression of transporter-encoding genes, 94 transport reactions were included in the network. To this end, the enhanced genome-scale metabolic network of *C. militaris* consisted of 2,929 genes, 714 EC numbers, 947 metabolites, 1,419 biochemical reactions and 94 transport reactions, which are summarized in Table 3. The enhanced genome-scale metabolic network of *C. militaris* is provided in Supplementary File 5 and was used for integrative analysis of transcriptome data.

**Table 3:** Statistical characteristics of enhanced genome-scale metabolic network.

Characteristics of metabolic network	This study	*i*WV1170^11^
Enzymes-encoding genes	2,929	1,170
Enzymes	714	679
Metabolites	947	894
Biochemical reactions	1,419	1,267
- Plasma membrane	89	87
- Cytosol	916	868
- Mitochondria	244	241
- Peroxisome	29	28
- Extracellular space	47	43
Transport reactions	94	0
Reactions with gene assignments	1,383	1,226
Reactions without gene assignments (gap)	36	48

### Identification of reporter metabolites and metabolic subnetworks associated with cordycepin production

To analyze the global metabolic response to carbon source alterations in cultivation, i.e., sucrose versus glucose condition, we applied the reporter metabolite algorithm to identify reporter metabolites and search for highly correlated metabolic subnetworks for pairwise carbon source comparisons^16,41^. This analysis relied on the enhanced genome-scale metabolic network of *C. militaris* (Supplementary File 5), and therefore, this analysis demonstrated how the metabolic network can be used to map the global regulatory response of *C. militaris*. The top 10 significant reporter metabolites affected by transcriptional up-regulation in response to sucrose compared to glucose cultures are listed in Table 4. Concerning cordycepin production, these metabolites made sense biologically since the identified reporter metabolites were related to all possible main precursors and intermediate metabolites in cordycepin biosynthesis, including adenosine (ADN), adenosine-3′-monophosphate (3AMP), 2′-carbonyl-3′-deoxyadenosine (2C3DA), adenosine triphosphate (ATP), adenosine-5′-monophosphate (AMP), S-adenosyl-L-methionine (SAM), S-adenosyl-L-homocysteine (SAH), 6-hydroxy-2-octaprenylphenol (2N6H), cordycepin (3DA) and aspartate (ASP). Aspartate was of interest because it was found to be a key metabolite involved in SAICAR related to cordycepin biosynthesis^42,43^.

**Table 4:** Significant reporter metabolites affected by transcriptional up-regulation in response to sucrose compared to glucose cultures.

Reporter metabolite	Distinct-directional p-value
Adenosine (ADN)	0.001996
Adenosine-3′-monophosphate (3AMP)	0.001996
2′-carbonyl-3′-deoxyadenosine (2C3DA)	0.001996
Adenosine triphosphate (ATP)	0.001996
Adenosine-5′-monophosphate (AMP)	0.001996
S-adenosyl-L-methionine (SAM)	0.001996
S-adenosyl-L-homocysteine (SAH)	0.001996
6-hydroxy-2-octaprenylphenol (2N6H)	0.001996
Cordycepin (3DA)	0.003992
Aspartate (ASP)	0.005988

**Figure 6:**
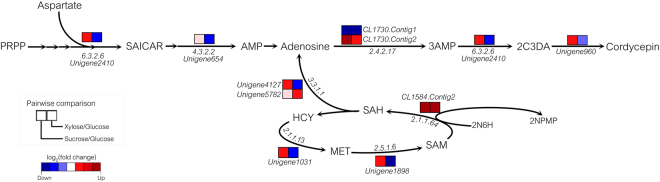
Key metabolic subnetworks of adenosine, cordycepin and methionine identified in *C. militaris*. Abbreviated metabolites are as follows: PRPP, phosphoribosyl pyrophosphate; AMP, adenosine-5′-monophosphate; 3AMP, adenosine-3′-monophosphate; SAICAR, phosphoribosylamino-imidazole-succinocarboxamide; 2N6H, 6-hydroxy-2-octaprenylphenol; SAM, S-adenosyl-L-methionine; SAH, S-adenosyl-L-homocysteine; HCY, homocysteine; MET, methionine; 2NPMP, 6-methoxy-2-octaprenylphenol; 2C3DA, 2′-carbonyl-3′-deoxyadenosine.

To further identify significant metabolic subnetworks, gene set enrichment analysis using the set of genes and their corresponding reactions from the enhanced metabolic network of *C. militaris* was performed. Figure 6 shows the key genes encoding enzymes/proteins and reporter metabolites that were identified in the significant metabolic subnetworks of *C. militaris* due to a change in carbon source, i.e., sucrose versus glucose and xylose versus glucose. Apparently, two significant metabolic subnetworks were identified, namely, the adenosine and cordycepin subnetwork and the methionine subnetwork. Considering the adenosine and cordycepin subnetwork, three enzymes/protein-encoding genes, including oxidoreductase domain-containing protein (Unigene960), SAICAR synthase, EC: 6.3.2.6 (Unigene2410) and ATP phosphoribosyltransferase, EC: 2.4.2.17 (CL1730.Contig2), which catalyze the transitional reactions towards the cordycepin biosynthetic pathway, showed significant transcriptional changes and were up-regulated in response to cordycepin production in sucrose culture. In contrast, the down-regulated genes associated with the adenosine and cordycepin subnetwork were mostly identified in xylose culture. Unexpectedly, a gene (CL1730.Contig2) encoding ATP phosphoribosyltransferase, EC: 2.4.2.17 was highly expressed in xylose culture. This finding suggests that ATP phosphoribosyltransferase may have different functional roles in the biosynthesis of other metabolites. Xia *et al*.^40^ found that ATP phosphoribosyltransferase was associated with pentostatin biosynthesis when PRPP and adenosine were used as substrates.

Surprisingly, the methionine subnetwork was also found to be related to cordycepin production. Three enzyme-encoding genes of interest were up-regulated in sucrose culture, namely, adenosyl homocysteinase, EC: 3.3.1.1 (Unigene4127); SAM synthase, EC: 2.5.1.6 (Unigene1898); and 3-demethylubiquinone-9 3-O-methyltransferase, EC: 2.1.1.64 (CL1584.Contig2). Consistently, these enzymes catalyzed the bioconversion of metabolites, i.e., 2N6H, SAM, and SAH, as shown in the list of reporter metabolites (Table 4).

Accordingly, the identification of reporter metabolites and metabolic subnetworks revealed key metabolic targets regarded as hot spots in the global metabolic regulation of the cordycepin biosynthetic pathway in *C. militaris*.

## Conclusion

Transcriptome analysis and genome-scale network-driven analysis highlighted the global metabolic responses in specific culture conditions during cordycepin overproduction in *C. militaris*.

## Methods

### Fungal cultivation, growth and cordycepin measurement

The fungal strain used in this study was *C. militaris* strain TBRC6039. The stock culture was maintained as a frozen mycelial suspension in 10% (v/v) glycerol at −80 °C^44^. For inoculum preparation, 0.5 mL of stock solution was added in a 250-mL shake-flask containing 75 mL of yeast extract-peptone-dextrose (YPD) medium, and the culture was grown at 22 °C with shaking at 250 rpm for 7 days without light induction. For cultivation, 5% (v/v) *C. militaris* inoculum was transferred into individual sterile 250-mL jars containing 75 mL of culture medium enclosed with a sterile filter cap. The medium was composed of 0.5 g/L MgSO_4_·7H_2_O, 0.5 g/L K_2_HPO_4_·3H_2_O, 0.5 g/L KH_2_PO_4_, 0.1 g/L CaCl_2_, 0.1 g/L FeSO_4_·7H_2_O, 40 mM (NH_4_)_2_SO_4_ and 20 g/L carbon source. The carbon sources used were sucrose, glucose and xylose. The cultivation was performed at 22 °C under static conditions for 60 days without light induction. All batch experiments were independently conducted in three biological replicates. The experimental setup from the cultivation process to transcriptome generation is illustrated in Fig. 1A. For dry cell weight (DCW) determination, mycelial cells were harvested through a filter paper, washed at least three times with distilled water and then dried using a freeze dryer at −110 °C until a constant weight was obtained. A portion of culture broth was used to analyze the cordycepin concentration by high-performance liquid chromatography (HPLC, Agilent, USA) in which the mobile phase was methanol/distilled water (15:85, v/v). Elution was performed at a flow rate of 1.0 mL/min on a HiQSil C18HS column (300 mm × 4.6 mm, 5 μm) at 40 °C using a UV detector with a wavelength of 260 nm. The residual sugar concentration was determined by HPLC (Thermo Scientific, USA). The mobile phase was 5 mmol/L H_2_SO_4_. Elution was performed at a flow rate of 0.6 mL/min on an Aminex HPX-87H column (300 mm × 7.8 mm) at 60 °C using an RI detector.

### RNA extraction and transcriptome sequencing

Mycelial cells were harvested at mid-log phases of sucrose, glucose and xylose cultures grown for 14, 16 and 45 days, respectively. Then, the harvested cells were immediately immersed in liquid nitrogen for RNA extraction. Up to 100 mg of mycelial samples were then ground using a cold mortar and pestle, and then, RNA extraction and purification was performed using an RNeasy mini kit (Qiagen) according to the instruction manual. The yield of RNA was assessed by measuring absorbance at 260 and 280 nm, which was followed by electrophoresis on a 1% (w/v) agarose gel (Bio-Rad, USA). The quality of total RNA was determined using an Agilent 2100 bioanalyzer and NanoDrop Spectrophotometer. Good quality total RNA was treated with DNase I to remove genomic DNA contamination, and then, magnetic beads coated with oligodeoxythymidylate (oligo(dT)) primers were used for mRNA isolation. For construction of the sequencing library, mRNA was mixed with the fragmentation buffer, and then the fragmented mRNAs were used as templates for cDNA library synthesis. The library was subsequently connected with paired-end adaptors and sequenced in parallel mode on the Illumina HiSeq. 4000 sequencer. RNA-Seq data, called raw data or raw reads were kept in FASTQ format^45^. The FASTQ files, including detailed read sequences and read quality information for each library, were deposited in NCBI Sequence Read Archive (SRA) under the BioProject accession number PRJNA416937.

### *De novo* transcriptome assembly and assessment

Based on the sequencing quality assessment, ambiguous reads that had more than 5% unknown bases (“N”) or a sequencing quality was less than 20% per read were filtered out using in-house software developed by Beijing Genome Institute (BGI). The remaining reads with a length of 100 base pairs (bps), called clean reads, were then proceeded through the Trinity reconstruction pipeline to generate full-length transcripts^15^. The overall transcriptome assembly and assessment procedure is illustrated in Fig. 1B. Briefly, clean reads were assembled into contigs based on a k-mer overlap by the Inchworm module. The assembled contigs were clustered and partitioned into many de Bruijn graphs individually based on clean read sharing contigs by the Chrysalis module. The individual graph extracted all possible sequences for extending contig lengths, and contig linkages were later determined from these sequences based on the available paired-end data by the Butterfly module. The extending contigs were incorporated into scaffolds and finally called transcripts. Then, the clean reads were mapped back to the Trinity-assembled transcript using Bowtie software^46^. Expression abundances were estimated as FPKM using RNA-Seq by Expectation-Maximization (RSEM) software^47,48^. Trinity transcripts were divided into two groups, i.e., cluster genes (CL-genes) and singleton genes (unigenes), based on non-redundant genes by gene family clustering analysis using TIGR gene indices clustering tools (TGICL) software^49^. If several transcripts shared 70% sequence similarity (isoform), they were classified into the CL-genes group and were given the prefix “CL”. Otherwise, they were classified into the singleton genes group with the prefix “Unigene”. The genes from all libraries were pooled and clustered again to obtain the final non-redundant gene set. Next, the completeness of gene representation in the transcriptome was tested by a homology search against the dataset of core eukaryotic genes (CEGs), which was shown to be a reliable indicator of completeness of gene space in eukaryotic species^50^. Typically, gene sequences that were smaller than the size selection cut-off of 200 bps were excluded^51^.

### Functional annotation and protein sequence comparisons

BLASTX analysis^52^ was performed to obtain protein-encoding genes from the transcriptome data by searching the gene sequences as queries against the functional protein databases, e.g., NCBI non-redundant protein sequence database (NR) (ftp://ftp.ncbi.nlm.nih.gov/blast/db), KEGG^25^, SwissProt^20^, Gene Ontology (GO)^21,22^, cluster of orthologous groups (COGs)^23^ and protein sequence analysis and classification (InterPro)^24^, using an E-value of 10^−5^ as a cut-off. The remaining gene sequences were subjected to the ESTScan program^26^ to detect additional protein-encoding genes. In addition, the protein-encoding genes identified from the *C. militaris* strain TBRC6039 transcriptome and published protein sequences from the *C. militaris* strain Cm01 genome^12^ deposited in the MycoCosm portal^53^ were compared against each other by using BLASTP to identify their homologues. An estimated expectation cut-off of 10^−50^, alignment length of 200 amino acids and percentage identity of 50% were applied to evaluate the statistical significance of conserved homologues. Bidirectional BLASTP was then applied to obtain a conservative set of 1:1 homologues between the two genomes of the *C. militaris* strains.

### Differential gene expression, clustering analysis and transcription factor identification

Significant DEGs were defined by altered expression levels with a fold change ≥2 and FDR ≤ 0.001 according to the previous report^54^. Using this method, the significant DEGs were identified across different carbon sources cultures through a pairwise comparison analysis^55^. The expression profiles of significant DEGs were then clustered by Pearson correlation under a complete-linkage hierarchical clustering method using the hclust and cutree functions from R base stats package. For visualization of the gene cluster, a heat map diagram was generated by using the heatmap.2 function in the R gplots package^56^. For transcription factor identification, the promoter region of each gene in a different cluster was retrieved from the MycoCosm portal^53^, and then DREME^57^ was used to search for DNA motifs with an E-value of 10^−5^ as a cut-off. To this end, transcription factors were identified by finding candidate transcription factor binding sites via sequence matching to DNA motifs within the promoter region using the Tomtom algorithm^58^ and JASPAR 2018 CORE collection^59^.

### Enhancement of genome-scale network reconstruction and reporter metabolite analysis

The identified protein-encoding genes from the *C. militaris* strain TBRC6039 transcriptome were then incorporated into the genome-scale metabolic network of *C. militaris* (*i*WV1170)^11^ based on their corresponding gene-protein-reaction (GPR) associations. According to the functional annotation, newly identified GPR associations of protein-encoding genes, which were obtained from homology searches, biochemical textbooks and the literature, were manually included in the network. To further enhance the metabolic reconstruction, genes encoding for transporters and their associated reactions from the transporter classification database (TCDB)^60^ were used to improve network connectivity.

Once the reconstructed genome-scale network was enhanced, it could be used as a scaffold for reporter metabolite analysis. This analysis was performed using gene level statistics (e.g., p-value), a set of genes and their associated metabolites as the inputs^16,41^. If a metabolite had a distinct-directional p-value below 0.01 (pDistinctDirUp or pDistinctDirDn in the R package Piano)^41^, then it was identified as a significant reporter metabolite. In a similar manner, a set of genes and their corresponding reactions associated with reporter metabolites was considered to be a significant metabolic subnetwork.

### Data accessibility

Raw Illumina HiSeq. 4000 sequences have been deposited to NCBI Sequence Read Archive (SRA) under the BioProject accession number PRJNA416937.

## Electronic supplementary material

Dataset 1

Dataset 2

Dataset 3

Dataset 4

Dataset 5
